# OLDER ADULTS’ EXPERIENCES WITH INNOVATIONS IN HEALTH CARE DELIVERY: FINDINGS FROM THE NPHA AND AARP SURVEYS

**DOI:** 10.1093/geroni/igae098.0083

**Published:** 2024-12-31

**Authors:** Erica Solway, Teresa Keenan, Teresa Keenan

**Affiliations:** Chair; Co-Chair; Discussant

## Abstract

Innovations in health care delivery have resulted in dramatic shifts in the experience of seeking and receiving health care services. Many of these innovations, like telehealth and patient portals, predated the COVID-19 pandemic, but the pandemic quickly spurred rapid changes in availability and use. It is important to understand how older adults view and experience these innovations and the opportunities and challenges these innovations present. Since its launch in 2017, the National Poll on Healthy Aging (NPHA) has surveyed a nationally representative sample of adults ages 50-80 to gauge their perspectives on health care and health policy-related topics, including health care innovations, and assess trends and changes in views and experiences over time. In addition, AARP regularly conducts surveys to offer additional timely insights on important related topics. This symposium will focus on lessons learned from the NPHA related to telehealth, patient portals, direct-to-consumer health care services, and use of alternative sites for health care, like urgent care and retail clinics. In addition, it will highlight key findings from recent AARP surveys related to views on innovations in health care delivery among adults and older adults and will share how these insights can inform policy and practice.

